# Clinical and Genetic Risk Factors for Acute Incident Venous Thromboembolism in Ambulatory Patients With COVID-19

**DOI:** 10.1001/jamainternmed.2022.3858

**Published:** 2022-08-18

**Authors:** JunQing Xie, Albert Prats-Uribe, Qi Feng, YunHe Wang, Dipender Gill, Roger Paredes, Dani Prieto-Alhambra

**Affiliations:** 1 Centre for Statistics in Medicine and National Institute for Health and Care Research Biomedical Research Centre Oxford, Nuffield Department of Orthopaedics, Rheumatology and Musculoskeletal Sciences, University of Oxford, Oxford, England; 2 Nuffield Department of Population Health, University of Oxford, Oxford, England; 3 Department of Epidemiology and Biostatistics, School of Public Health, Imperial College London, London, England; 4 Department of Clinical Pharmacology and Therapeutics, Institute for Infection and Immunity, St George's, University of London, London, England; 5 Genetics Department, Novo Nordisk Research Centre Oxford, Old Road Campus, Oxford, England; 6 Infectious Diseases Department and irsiCaixa AIDS Research Institute, Hospital Universitari Germans Trias i Pujol, Catalonia, Spain

## Abstract

**Importance:**

The risk of venous thromboembolism (VTE) in ambulatory COVID-19 is controversial. In addition, the association of vaccination with COVID-19–related VTE and relevant clinical and genetic risk factors remain to be elucidated.

**Objective:**

To quantify the association between ambulatory COVID-19 and short-term risk of VTE, study the potential protective role of vaccination, and investigate clinical and genetic risk factors for post–COVID-19 VTE.

**Design, Setting, and Participants:**

This population-based cohort study of patients with COVID-19 from UK Biobank included participants with SARS-CoV-2 infection that was confirmed by a positive polymerase chain test reaction result between March 1, 2020, and September 3, 2021, who were then propensity score matched to COVID-19–naive people during the same period. Participants with a history of VTE who used antithrombotic drugs (1 year before index dates) or tested positive in hospital were excluded.

**Exposures:**

First infection with SARS-CoV-2, age, sex, ethnicity, socioeconomic status, obesity, vaccination status, and inherited thrombophilia.

**Main Outcomes and Measures:**

The primary outcome was a composite VTE, including deep vein thrombosis or pulmonary embolism, which occurred 30 days after the infection. Hazard ratios (HRs) with 95% CIs were calculated using cause-specific Cox models.

**Results:**

In 18 818 outpatients with COVID-19 (10 580 women [56.2%]; mean [SD] age, 64.3 [8.0] years) and 93 179 matched uninfected participants (52 177 women [56.0%]; mean [SD] age, 64.3 [7.9] years), the infection was associated with an increased risk of VTE in 30 days (incidence rate of 50.99 and 2.37 per 1000 person-years for infected and uninfected people, respectively; HR, 21.42; 95% CI, 12.63-36.31). However, risk was substantially attenuated among the fully vaccinated (HR, 5.95; 95% CI, 1.82-19.5; interaction *P* = .02). In patients with COVID-19, older age, male sex, and obesity were independently associated with higher risk, with adjusted HRs of 1.87 (95% CI, 1.50-2.33) per 10 years, 1.69 (95% CI, 1.30-2.19), and 1.83 (95% CI, 1.28-2.61), respectively. Further, inherited thrombophilia was associated with an HR of 2.05 (95% CI, 1.15-3.66) for post–COVID-19 VTE.

**Conclusions and Relevance:**

In this population-based cohort study of patients with COVID-19, ambulatory COVID-19 was associated with a substantially increased risk of incident VTE, but this risk was greatly reduced in fully vaccinated people with breakthrough infection. Older age, male sex, and obesity were clinical risk factors for post–COVID-19 VTE; factor V Leiden thrombophilia was additionally associated with double the risk, comparable with the risk of 10-year aging. These findings may reinforce the need for vaccination, inform VTE risk stratification, and call for targeted VTE prophylaxis strategies for unvaccinated outpatients with COVID-19.

## Key Points

**Question:**

What is the 30-day acute risk of venous thromboembolism (VTE) among ambulatory patients with COVID-19, and what are the clinical and genetic risk factors predisposing them to developing post–COVID-19 VTE?

**Findings:**

In this retrospective cohort study of 18 818 outpatients with COVID-19 and 93 179 propensity score–matched noninfected participants, a higher VTE incidence was observed in the former (hazard ratio, 21.42); however, this risk was considerably attenuated among the fully vaccinated, after breakthrough infection. Older age, male sex, obesity, no vaccination or partial vaccination, and inherited thrombophilia were independent risk factors for COVID-19–associated VTE.

**Meaning:**

The results of this study suggest that ambulatory patients with COVID-19, either vaccinated or not, present a clinically relevant increased risk of incident VTE during the acute phase, with the risk pronounced by factors of older age, male sex, obesity, incomplete vaccination, and factor V Leiden thrombophilia.

## Teaser

This cohort study examines the association between ambulatory COVID-19 and risk of venous thromboembolism.

## Introduction

Numerous hospital-based studies and case series have demonstrated a high risk of venous thromboembolism (VTE) in patients with severe COVID-19. A recent meta-analysis reported a pooled VTE rate of 14.7% and 23.2% among those admitted to the hospital and intensive care units, respectively.^1^ Additionally, emerging randomized clinical trials^2,3,4^ have shown the benefit-risk of anticoagulation for patients with COVID-19 at different stages of the disease, and clinical societies have recommended initiating routine antithrombotic therapy during hospital admission.^5^

In contrast, fewer clinical interventions have been implemented to prevent VTE among ambulatory patients with SARS-CoV-2 infection, partially because of conflicting findings on the association between the infection and VTE occurrence, with cohort studies showing no elevated risk^6^ but self-controlled case series studies consistently showing a substantial increase of short-term risk.^7,8,9^ Also, mixed evidence of benefit from oral anticoagulation complicated clinical decisions for ambulatory COVID-19.^10^ Given the ongoing global rollout of vaccines, relaxation of public health restrictions, and the widespread highly transmissible Omicron variant, the absolute number of milder COVID-19 cases treated in ambulatory settings continues to rise worldwide.^11,12^ These collectively suggest that prophylaxis, including timing and dosing regimens, requires further refinement, particularly in the outpatient setting.^2,3,4,13,14^ Moreover,^15^ a lack of insight into the association of clinical, socioeconomic, and genetic risk factors with infection-related VTE persists. This study aimed to (1) quantify the magnitude of short-term VTE risk associated with SARS-CoV-2 infection identified in ambulatory settings and (2) investigate the clinical and genetic risk factors for VTE after SARS-CoV-2 infection.

## Methods

We included UK Biobank (UKBB) participants from England who were alive on March 1, 2020. All participants provided written informed consent at the UKBB cohort recruitment. This study followed the Strengthening the Reporting of Observational Studies in Epidemiology (STROBE) reporting guidelines and received ethical approval from the UKBB ethics advisory committee.

### Data Sources and Study Cohorts

We obtained data from UK Biobank comprising multiple linked sources, including baseline surveys conducted between 2006 and 2010, individual genetic data, primary care electronic medical records, hospital inpatient data from Hospital Episode Statistics, diagnostic COVID-19 test data from the Public Health England's Second Generation Surveillance System,^16^ and death records from the national death registry (Office of National Statistics).

 We curated an infected cohort by enrolling individuals with positive polymerase chain reaction SARS-CoV-2 test results that were confirmed between March 1, 2020, and September 30, 2021. Participants who were never tested or only had negative test results were classified into the noninfected cohort. The index date was the date of the first positive specimen sample for the infected cohort. A random date that followed the same calendar period distribution of the index date as the infected individuals was assigned to the noninfected individuals. Participants with a history of VTE or who used oral anticoagulants and antiplatelet drugs 1 year before the index dates were excluded. Additionally, we excluded those in the infected cohort who were already hospitalized at the time of testing positive for COVID-19. Any information after the index date was not used for the cohort exclusion (eFigure 1 in the Supplement).

### Inherited Thrombophilia

Information on genotyping and imputation procedures in UK Biobank has been detailed in previous studies.^17^ Briefly, genome-wide single-nucleotide polymorphisms (SNPs) were genotyped using 2 closely related purpose-designed arrays (the UK BiLEVE Axiom array and UK Biobank Axiom array). We defined inherited thrombophilia carriers as having any of 2 risk SNP variants in factor V Leiden (rs6025) or prothrombin G20210A (rs1799963). We also defined a positive genetic control exposure by calculating a 297-SNPs polygenic risk score (PRS) for VTE that did not include these 2 variants^18^ (eMethods in the Supplement).

### Covariates

We prespecified a list of covariates for adjustment based on clinicians’ knowledge, including demographic characteristics (age, sex, self-reported race and ethnicity grouped into other racial and ethnic categories that included Asian or Asian British, Black or Black British, Chinese, and unspecific/unknown ethnicity or White, given that approximately 90% of the UKBB participants were White), socioeconomic status measured by the Index of Multiple Deprivation (a continuous summary deprivation measurement used in England containing 7 aspects in crime, education, employment, health, housing, income, and living environment),^19^ body mass index (BMI), medications for chronic illness prescribed within 1 year before the index date, and trauma-related diagnosis and all comorbidities included in the Charlson Comorbidity Index (Table 1).^20^ The orthopedic surge, number of hospital admissions during the past year (proxy of health care utilization), and vaccination status (not or partially vaccinated vs fully vaccinated) were also studied.

**Table 1.** Baseline Characteristics of Participants Stratified by the SARS-CoV-2 Infection Status Before and After Matching^a^

Characteristic	Unmatched cohorts			Matched cohorts		
	Uninfected	Infected	SMD^b^	Uninfected	Infected	SMD^b^
No.	317 943	18 847	NA	93 179	18 818	NA
Age, mean (SD)	67.96 (8.03)	64.32 (8.03)	0.453	64.31 (7.92)	64.33 (8.03)	0.002
Sex, No. (%)						
Female	185 897 (58.5)	10 600 (56.2)	0.045	52 177 (56.0)	10 580 (56.2)	0.005
Male	132 046 (41.5)	8247 (43.8)	0.045	41 002 (44.0)	8238 (43.8)	0.005
Race and ethnicity, No. (%)						
White	297 595 (93.6)	16 590 (88.0)	0.194	83 261 (89.4)	16 588 (88.1)	0.038
Multiracial/unknown^c^	20 348 (6.4)	2257 (12.0)	0.194	9918 (10.6)	2230 (11.9)	0.038
Index of multiple deprivation, mean (SD)	17.03 (13.51)	19.87 (14.68)	0.201	19.41 (14.92)	19.83 (14.65)	0.028
Body mass index, mean (SD)^d^	27.04 (4.61)	27.65 (4.83)	0.130	27.58 (4.99)	27.64 (4.81)	0.014
Vaccination status, No. (%)						
Not or partially vaccinated	195 574 (61.5)	11 156 (59.2)	0.047	55 183 (59.2)	11 135 (59.2)	0.001
Fully vaccinated	122 369 (38.5)	7691 (40.8)	0.047	37 996 (40.8)	7683 (40.8)	0.001
Recent medications, No. (%)						
Lipid-lowering drugs	87 164 (27.4)	4634 (24.6)	0.064	22 507 (24.2)	4627 (24.6)	0.010
RAS inhibitors	57 832 (18.2)	3296 (17.5)	0.018	16 035 (17.2)	3292 (17.5)	0.008
Other antihypertensives	25 961 (8.2)	1367 (7.3)	0.034	6750 (7.2)	1363 (7.2)	0.001
Proton pump inhibitors	78 080 (24.6)	5263 (27.9)	0.077	25 371 (27.2)	5246 (27.9)	0.015
Diabetes medicines	16 108 (5.1)	1112 (5.9)	0.037	5238 (5.6)	1106 (5.9)	0.011
Antidepressants	43 310 (13.6)	3188 (16.9)	0.092	15 483 (16.6)	3178 (16.9)	0.007
Systemic glucocorticoids	14 237 (4.5)	972 (5.2)	0.032	4623 (5.0)	969 (5.1)	0.009
Immunosuppressants	3541 (1.1)	203 (1.1)	0.004	938 (1.0)	203 (1.1)	0.007
Antineoplastic agents	178 (0.1)	14 (0.1)	0.007	66 (0.1)	14 (0.1)	0.001
Recent orthopedic surgery, No. (%)	6018 (1.9)	371 (2.0)	0.006	1844 (2.0)	371 (2.0)	0.001
Recent hospital admissions, mean (SD)	0.29 (1.48)	0.31 (1.95)	0.014	0.30 (1.61)	0.31 (1.95)	0.011
Coexisting conditions, No. (%)						
Fracture	57 539 (18.1)	3415 (18.1)	0.001	16 850 (18.1)	3414 (18.1)	0.002
Fall	24 063 (7.6)	1381 (7.3)	0.009	6721 (7.2)	1374 (7.3)	0.003
Cancer	31 271 (9.8)	1476 (7.8)	0.071	7175 (7.7)	1474 (7.8)	0.005
Malignant cancer	1119 (0.4)	63 (0.3)	0.003	297 (0.3)	63 (0.3)	0.003
Diabetes (uncomplicated)	23 250 (7.3)	1496 (7.9)	0.024	7203 (7.7)	1490 (7.9)	0.007
Diabetes (end-organ damage)	6714 (2.1)	415 (2.2)	0.006	2004 (2.2)	415 (2.2)	0.004
Congestive heart failure	1666 (0.5)	89 (0.5)	0.007	445 (0.5)	89 (0.5)	0.001
Myocardial infarction	658 (0.2)	34 (0.2)	0.006	161 (0.2)	34 (0.2)	0.002
Cerebrovascular disease	2500 (0.8)	138 (0.7)	0.006	655 (0.7)	138 (0.7)	0.004
Peripheral vascular disease	1379 (0.4)	49 (0.3)	0.030	213 (0.2)	49 (0.3)	0.006
Liver disease (mild)	1506 (0.5)	81 (0.4)	0.007	401 (0.4)	80 (0.4)	0.001
Liver disease (moderate to severe)	607 (0.2)	46 (0.2)	0.011	231 (0.2)	46 (0.2)	0.001
COPD	49 834 (15.7)	3309 (17.6)	0.051	16 182 (17.4)	3302 (17.5)	0.005
Chronic kidney disease	12 847 (4.0)	691 (3.7)	0.019	3367 (3.6)	690 (3.7)	0.003
Peptic ulcer	6256 (2.0)	419 (2.2)	0.018	2039 (2.2)	414 (2.2)	0.001
Rheumatoid arthritis	8363 (2.6)	511 (2.7)	0.005	2412 (2.6)	510 (2.7)	0.008
Dementia	1902 (0.6)	205 (1.1)	0.054	998 (1.1)	202 (1.1)	0.001
Hemiplegia	242 (0.1)	15 (0.1)	0.001	83 (0.1)	15 (0.1)	0.003
AIDS	309 (0.1)	17 (0.1)	0.002	76 (0.1)	17 (0.1)	0.003

Abbreviations: AIDS, acquired immune deficiency syndrome; COPD, chronic obstructive pulmonary disease; NA, not applicable; SMD, standardized mean difference.

^a^ The look-back window of covariates for recent medications, orthopedic surgery, and hospital admissions was past 1 year before the index date and for coexisting conditions was any time before the index date. Age, index of multiple deprivation, body mass index, and recent hospital admissions were included as the continuous and other covariates as binary in logistic regression model for the generation of propensity score.

^b^ Value ≤0.10 is considered good balance.

^c^ Includes Asian or Asian British, Black or Black British, Chinese, and unspecific/unknown ethnicity.

^d^ Calculated as weight in kilograms divided by height in meters squared.

### Outcomes

Incident VTE, comprising either deep vein thrombosis or pulmonary embolism, was identified using *International Statistical Classification of Diseases and Related Health Problems, Tenth Revision (ICD-10)* codes based on hospital records. Eligible participants were followed up for up to 30 days after the index date, given that VTE occurring after 30 days was much less likely to be associated with SARS-CoV-2 infection.

### Statistical Analyses

We used propensity score (PS) matching to minimize confounding in studying the association between SARS-CoV-2 infection and VTE. We fitted multivariable logistic regression models to estimate PS as the probability of infection based on all predefined covariates. We then matched infected with noninfected individuals with a ratio of 1:5 based on PS values, with a caliper width of up to 0.2 standard deviations of the logit of the PS, with exact matching on index dates.^21,22^ We assessed the covariate balance between the cohorts before and after matching using absolute standardized mean differences (SMDs) and specified an SMD greater than 0.1 as relevant imbalances.^23^ Cause-specific Cox survival models were applied to estimate the hazard ratio (HR) for VTE according to exposure, for which death was considered a competing risk.^24^ The overall HR and that in subgroups by prior vaccination status were provided. The multiplicative interaction effect between the infection and vaccination status was tested statistically on the probability scale.

To study clinical risk factors, we fitted multivariable Cox models of 30-day VTE in the ambulatory COVID-19 cohort, including age, sex, race and ethnicity, socioeconomic status, obesity (BMI less than vs equal or more than 30), vaccination status, cancer, fall, fracture, and the number of comorbidities. For the analysis of the association of inherited thrombophilia with post–COVID-19 VTE, we adjusted Cox models for age, sex, and genetic ancestry (quantified by the first 3 principal components), assuming that genetic variants were independent of all other baseline characteristics.

We performed a sensitivity analysis repeating the modeling of clinical risk factors with VTE in the uninfected cohort, in which no association was expected between vaccination and COVID-19–unrelated VTE. We also introduced a positive exposure control (PRS for VTE) and a negative outcome (arterial thromboembolism) experiment to detect residual confounding and potential unresolved bias for the exposure of inherited thrombophilia.^25^

All statistical tests were 2-sided, for which a *P* = .05 or a 95% CI that did not cross 1 were considered statistically significant for the primary analyses. All analyses and data visualizations were conducted using R, version 4.1.2 (R Foundation). Genetic data management and quality controls were performed using Plink 1.9.^26^

## Results

### Baseline Characteristics

Out of 407 311 UKBB participants, 26 210 (6.4%) had SARS-CoV-2 infection between March 1, 2020, and September 30, 2021. After applying exclusion criteria, 21 724 of 26 210 (83.0%) infected and 317 943 of 380 398 (83.5%) noninfected individuals were eligible for analyses (eFigure 1 in the Supplement). For all infections, 2877 (13.2%) and 18 847 (86.8%) were tested in hospital and outpatient settings, respectively. Only the latter were included for subsequent analyses.

Baseline characteristics by infection status and incident VTE outcome are summarized in Table 1 and eTable 1 in the Supplement. Before matching, ambulatory participants with COVID-19 were younger than those without infection (mean [SD] age, 64.32 [8.03] vs 67.96 [8.03]), more likely male (8247 [43.8%] vs 132 046 [41.5%]), of a racial and ethnic minority group (2257 [12.0%] vs 20 348 [6.4%]), experienced greater socioeconomic deprivation, and had obesity. After 1:5 PS matching, 18 818 patients with COVID-19 were matched to 93 179 uninfected counterparts (98.6% of COVID-19 with 5 matches). All covariates became balanced (eg, mean [SD] age of 64.31 [7.92] years and 44.0% male in the COVID-19 group vs mean [SD] age of 64.33 [8.03] years and 43.8% male in controls). In addition, index dates and calendar periods were accurately aligned between the cohorts, as depicted in eFigure 2 in the Supplement.

### Association of SARS-CoV-2 Infection With Incident VTE

**Figure 1.**
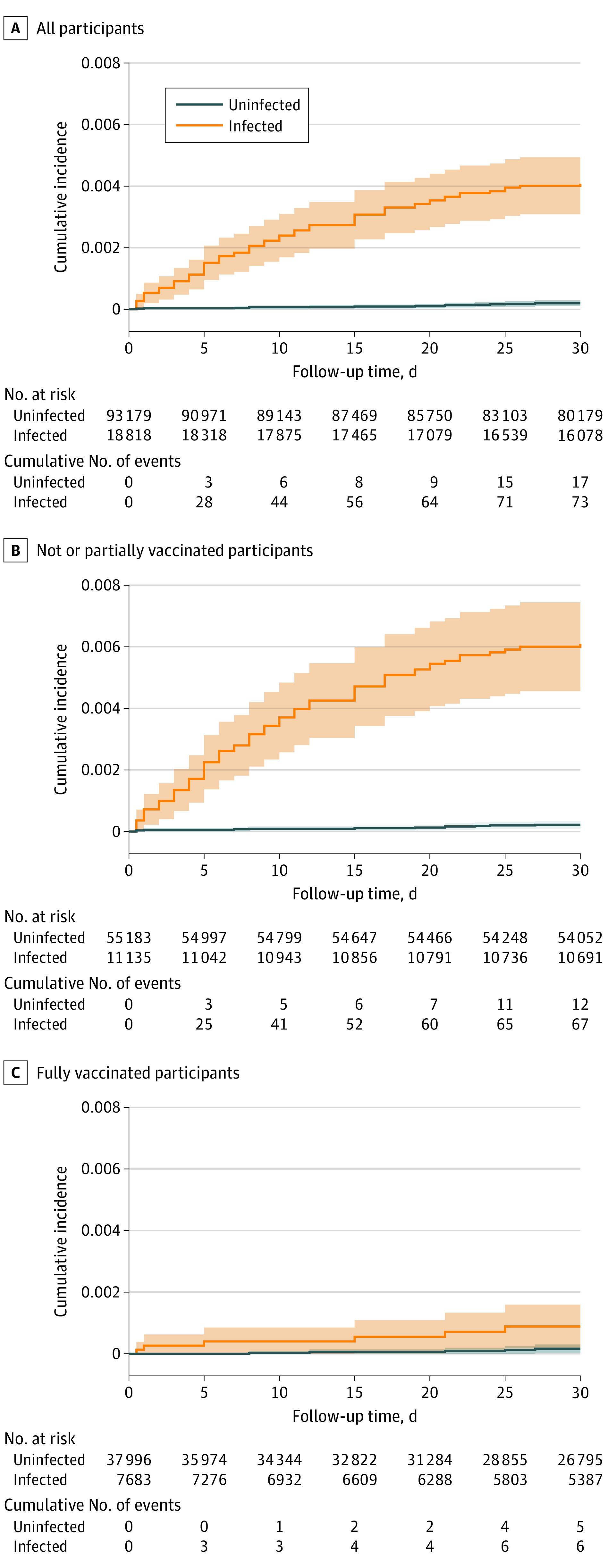
Cumulative Incidence Curves of Venous Thromboembolism Within 30 Days Overall and in Subgroups by Vaccination Status

Figure 1 depicts the cumulative incidence of VTE according to infection status, showing an early separation of the matched cohorts, with continued divergence over time. A total of 73 and 17 VTE events were seen within 30 days among the ambulatory patients with COVID-19 and matched uninfected individuals, which corresponded to incidence rates of 50.99 and 2.37 per 1000 person-years, respectively. Survival analyses (Table 2) suggested that the infection was associated with a substantial increase in VTE risk (HR, 21.42; 95% CI, 12.63-36.31). The observed risk was more pronounced in the unvaccinated patients (HR, 27.94; 95% CI, 15.11-51.65) and significantly mitigated in those fully vaccinated (HR, 5.95; 95% CI, 1.82-19.51; interaction *P* = .02).

**Table 2.** Associations Between Ambulatory COVID-19 and Venous Thromboembolism Overall and in Subgroups by Vaccination Status

Characteristic	Uninfected			Infected			Hazard ratio (95% CI)	*P* value for interaction
	No. of people	No. of events	Incidence per 1000 patient-y	No. of people	No. of events	Incidence per 1000 patient-y		
Overall	93 179	17	2.37	18 818	73	50.99	21.42 (12.63-36.31)	NA
Subgroups								
Not or partially vaccinated^a^	55 183	12	2.67	11 135	67	74.96	27.94 (15.11-51.65)	.02
Fully vaccinated	37 996	5	1.87	7683	6	11.15	5.95 (1.82-19.51)	

Abbreviation: NA, not applicable.

^a^ A total of 92.2% of people were not vaccinated, and 7.8% were partially vaccinated.

### Clinical Determinants of Post–COVID-19 VTE

**Figure 2.**
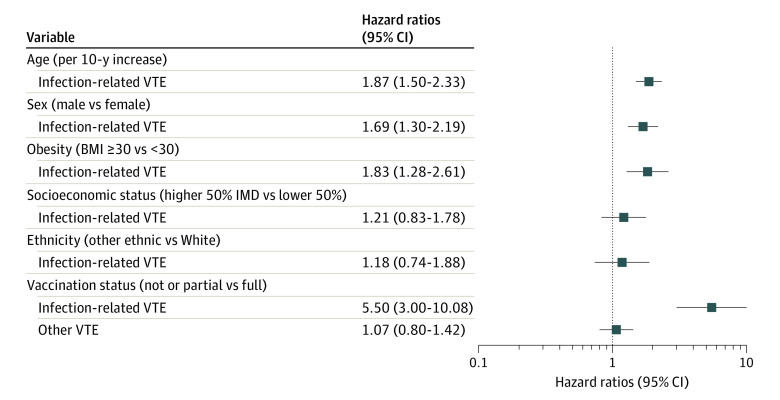
Hazard Ratio of Clinical Risk Factors for Venous Thromboembolism (VTE) Among Patients With COVID-19 Covariates included in the single multivariable Cox regression model were age, sex, race and ethnicity, socioeconomic status, obesity, vaccination status, cancer, fall, fracture, and number of comorbidities. The estimate of hazard ratios for other VTE was calculated among the uninfected group, which was predefined as a negative control outcome analysis. BMI indicates body mass index (calculated as weight in kilograms divided by height in meters squared); IMD, index of multiple deprivation.

The associations between sociodemographic and clinical factors (including vaccination status) and the risk of post–COVID-19 VTE are shown in Figure 2. Older participants had a higher risk, with an approximate doubling of risk per each 10-year increase in age (adjusted HR, 1.87; 95% CI, 1.50-2.33). Men were at higher risk than women (adjusted HR, 1.69; 95% CI, 1.30-2.19), and people with obesity at a higher risk than non-obese (adjusted HR, 1.83; 95% CI, 1.28-2.61). These associations were similarly seen for COVID-19–unrelated VTE in direction and magnitude (eTable 2 in the Supplement).

Additionally, no or partial vaccination was associated with evident higher risk of COVID-19–related VTE (adjusted HR, 5.50; 95% CI, 3.00-10.08). A sensitivity analysis demonstrated no association between vaccination status and COVID-19–unrelated VTE among the uninfected participants, with an HR equal to 1.07 (95% CI, 0.80-1.42) (Figure 2; eTable 2 in the Supplement).

### Inherited Thrombophilia and Post–COVID-19 VTE

Among 21 055 infected participants with complete genetic data, 1287 (6.11%) had inherited thrombophilia, with 909 (4.32%) and 392 (1.86%) carrying risk variant/s of factor V Leiden and prothrombin G20210A, respectively (Table 3). The frequency of these genetic variations in the infected cohort was like that in the overall UKBB cohort and consistent with reports from previous literature (eTable 3 in the Supplement). No differences in any of the measured covariates (except for race and ethnicity), including sociodemographic characteristics, medications, or comorbidities, were observed when comparing those with vs without inherited thrombophilia (eTable 4 in the Supplement).

**Table 3.** Association of Inherited Thrombophilia With Venous and Arterial Thromboembolism Among Patients With COVID-19^a^

Exposure	Primary outcome, venous thromboembolism			Negative outcome, arterial thromboembolism		
	HR (95% CI)		*P* value	HR (95% CI)		*P* value
	Unadjusted	Adjusted		Unadjusted	Adjusted	
Inherited thrombophilia^b^	1.82 (1.02-3.23)	2.05 (1.15-3.66)	.01	0.94 (0.51-1.73)	0.98 (0.53-1.80)	.95
Factor V Leiden	1.97 (1.03-3.76)	2.17 (1.13-4.15)	.02	0.97 (0.48-1.98)	0.99 (0.49-2.01)	.97
Prothrombin G20210A^c^	1.31 (0.42-4.11)	1.52 (0.48-4.79)	.45	0.84 (0.27-2.62)	0.91 (0.29-2.84)	.86
Positive control						
Continuous PRS^d^	1.29 (1.08-1.54)	1.34 (1.11-1.60)	<.001	NC	NC	NC
Categorical PRS^e^	1.39 (0.73-2.66)	1.54 (0.80-2.95)	.20	NC	NC	NC

Abbreviations: HR, hazard ratio; NC, not calculated; PRS, polygenic risk score.

^a^ To set an informative positive exposure arm, we classified people into low and high polygenic risk groups according to their PRS value with a cutoff point at the 94th percentile. This threshold was chosen based on the proportion of inherited thrombophilia in the general UK Biobank population (approximately 6%).

^b^ Any risk variations in factor V Leiden and prothrombin G20210A.

^c^ Minimum detectable incidence rate ratio associated with the exposure of prothrombin G20210A is 3.58 less than the current sample size.

^d^ Per 1-SD increase of PRS.

^e^ Top 6% of PRS vs the lower.

Participants with inherited thrombophilia had a higher risk of VTE following SARS-CoV-2 infection than those without (adjusted HR, 2.05; 95% CI, 1.15-3.66). For each risk variant, the adjusted HR was 2.17 (95% CI, 1.13-4.15) for factor V Leiden carriers and 1.52 (95% CI, 0.48-4.79) for prothrombin G20210A carriers. Also, individuals with higher PRS values had greater VTE risk (adjusted HR per 1-SD increase of PRS, 1.33; 95% CI, 1.11-1.59) (Table 3). As expected, no associations were observed between inherited thrombophilia and the negative control outcome of arterial thromboembolism, with adjusted HRs ranging from 0.91 (95% CI, 0.29-2.84) to 0.99 (95% CI, 0.49-2.01).

## Discussion

In this community-based cohort study of UKBB participants, including 26 210 with polymerase chain reaction–confirmed ambulatory COVID-19 cases and 380 398 matched contemporary noninfected controls, we found that SARS-CoV-2 infection was associated with an increased VTE risk within 30 days of a positive test (HR, 21.42). However, this risk was largely attenuated in the fully vaccinated participants who then experienced a breakthrough infection (HR, 5.95). Known clinical risk factors for VTE, including older age, male sex, and obesity, applied to post–COVID-19 VTE. Also, compared with the full vaccination, no or partial vaccination was associated with an increased risk of post–COVID-19 VTE. As expected, vaccination was not associated with VTE risk in the uninfected peers. Finally, factor V Leiden thrombophilia carriers had an additional double risk of post–COVID-19 VTE compared with noncarriers, which was equivalent to the excess risk associated with an increase of 10 years of age.

The study finding of a substantially higher incidence of VTE in ambulatory COVID-19 disagreed with a previous meta-analysis of 7 heterogeneous small COVID-19 cohorts, which had suggested that mild COVID-19 was not a risk factor for VTE, with a combined overall relative risk equal to 1.18 (95% CI, 0.79-1.77).^6^ However, the present study’s data align more with extensive self-controlled case series studies^7,8,9^ that better accounted for within-person confounding and consistently showed orders of magnitude increases in VTE risk after the infection (eg, more than 46-fold and 7-fold higher risk at 7-14 days for pulmonary embolism and deep vein thrombosis, respectively).^7^ Most recently, a large network cohort study in 5 European countries profiled that 90-day incidence of VTE ranged from 0.2% to 0.8% among any patients with COVID-19 and up to 4.5% for those hospitalized.^27^

Public interest and concerns have been placed predominantly on vaccine-related rare thromboembolic events,^28^ which have been associated with vaccine hesitancy and restrictions on their use.^29^ The present study found that the vaccination may offset SARS-CoV-2–induced VTE risk even if people get a breakthrough infection. This evident benefit should not be ignored in the ongoing global vaccination campaigns.

To our knowledge, for the first time, we showed that inherited thrombophilia was associated with a double risk of COVID-19–related VTE, echoing previous clinical findings of elevated factor V activity in patients with severe COVID-19.^30,31,32^ A relatively high proportion of congenital thrombophilia was detected in a small pilot study of 87 COVID-19 cases, but the minimal sample size precluded further robust inference.^33^

We analyzed linked data that combined extensive community SARS-CoV-2 testing, well-recorded vaccination status, ambulatory and hospital-based clinical outcomes, and large-scale genotyping data that were readily available for UKBB participants. The results of this analysis have potentially noteworthy implications. First, VTE risk management needs reevaluation for milder ambulatory COVID-19. With emerging evidence and guidelines focusing on VTE prophylaxis for hospitalized patients with COVID-19, further work is necessary to mitigate the risk in the community. The recent ACTIV-4B^34^ study demonstrated no benefit of use of aspirin or apixaban for VTE prevention among the general younger outpatients (median age, 54 years [IQR, 46-59]), largely because of a very low thrombotic event rate. The present study of older participants (mean age, 64 years) might inform ongoing^35^ or new trials that target the more elderly population, particularly those with the multiple risk factors identified in this study. Second, although the etiology of post–COVID-19 VTE is complex and multifaceted, this study’s findings elucidated the role of factor V and possibly prothrombin proteins as contributing factors. Third, although genetic testing of inherited thrombophilia for VTE prevention has been previously discussed in many clinical scenarios,^36,37^ this newly identified association with COVID-19–related VTE, comparable with a 10-year aging risk, supports the potential value of targeted genetic screening for thrombophilia in the infected older adults. Finally, the study data suggest the significant association of vaccination with minimizing the risk of COVID-19 VTE.

### Limitations

This study also had some limitations. Residual confounding cannot be ruled out in this observational study, although robust statistical approaches for causal inference were applied, including PS matching and knowledge-driven negative control analyses. Differential outcome ascertainment might also have contributed to the elevated risk to some extent, given that SARS-CoV-2 infection since the pandemic was strongly believed to be a risk factor for VTE, and clinicians were probably more intentionally looking for VTE in patients with COVID-19 than others requiring hospital care. Although participants with COVID-19 were from nonhospital settings, they were tested likely because of the presence of typical symptoms of COVID-19. The extent to which purely asymptomatic infection is associated with VTE risk warrants further investigation. Also, the VTE in this study appeared to be clinically relevant events that trigger *ICD-10* coding. However, the diagnoses themselves did not necessarily reflect VTE status and severity (eg, asymptomatic, incidental, or symptomatic, which requires bespoke screening for VTE in patients with COVID-19). This study was performed among the antithrombotic use–naive population, and before the monoclonal antibody infusion or antivirals were approved for use; whether these treatments can mitigate ambulatory COVID-19–related VTE risk remains unclear. Moreover, the estimates from our analyses were an average and mixed short-term (30-day) effect of several SARS-CoV-2 strains from the original wild type to Delta,^38^ which should be cautiously extrapolated to ongoing or post–short-term periods and novel variants, such as Omicron. Finally, participants recruited in UKBB were not fully representative of the general population,^39,40^ and the data included in this study had very few participants who identified as Asian or Asian British, Black or Black British, or Chinese, which may limit the generalizability of the findings.

## Conclusions

In this population-based cohort study of patients with COVID-19, ambulatory COVID-19 was associated with a substantial increase in excess VTE. This risk was much higher among unvaccinated individuals and increased with older age, in men, and in patients with obesity. Factor V Leiden thrombophilia further doubled VTE risk, comparable with a 10-year aging risk. These findings call for targeted prevention and tailored thromboprophylaxis strategies for post–COVID-19 VTE in outpatient settings and suggest an etiological role of inherited thrombophilia.

## Notes

Supplement. eFigure 1. Flow-chart for the study design and participants’ eligibility eFigure 2. Distribution of infected and matched non-infected persons over the study period eTable 1. Baseline characteristics of participants stratified by the VTE outcome during the follow-up period eTable 2. Hazard ratio of clinical risk factors for venous thromboembolism amongs the infect and uninfected groups eTable 3. Frequency of risk variants for inherited thrombophilia eTable 4. Baseline characteristics amongst SARS-CoV-2 infected participants stratified by the inherited thrombophilia eMethods.
