# Supplementary material for: Clinical and Genetic Risk Factors for Acute Incident Venous Thromboembolism in Ambulatory Patients With COVID-19
Source: JAMA Intern Med. 2022 Aug 18;182(10):1063–71. doi: 10.1001/jamainternmed.2022.3858 (PMC9389434; doi:10.1001/jamainternmed.2022.3858)
Supplement: Supplement. — eFigure 1. Flow-chart for the study design and participants’ eligibility eFigure 2. Distribution of infected and matched non-infected persons over the study period eTable 1. Baseline characteristics of participants stratified by the VTE outcome during the follow-up period eTable 2. Hazard ratio of clinical risk factors for venous thromboembolism amongs the infect and uninfected groups eTable 3. Frequency of risk variants for inherited thrombophilia eTable 4. Baseline characteristics amongst SARS-CoV-2 infected participants stratified by the inherited thrombophilia eMethods. [file jamainternmed-e223858-s001.pdf]

Supplemental Online Content

Xie J, Prats-Uribe A, Feng Q, et al. Clinical and genetic risk factors for acute incident venous thromboembolism in ambulatory patients with COVID-19. *JAMA Intern Med*. Published online August 18, 2022. doi:10.1001/jamainternmed.2022.3858

eFigure 1. Flow-chart for the study design and participants' eligibility

eFigure 2. Distribution of infected and matched non-infected persons over the study period

eTable 1. Baseline characteristics of participants stratified by the VTE outcome during the follow-up period

eTable 2. Hazard ratio of clinical risk factors for venous thromboembolism amongs the infect and uninfected groups

eTable 3. Frequency of risk variants for inherited thrombophilia

eTable 4. Baseline characteristics amongst SARS-CoV-2 infected participants stratified by the inherited thrombophilia

eMethods.

This supplemental material has been provided by the authors to give readers additional information about their work.

© 2022 Xie J et al. *JAMA Internal Medicine.*

eFigure 1: Flow-chart for the study design and participants' eligibility

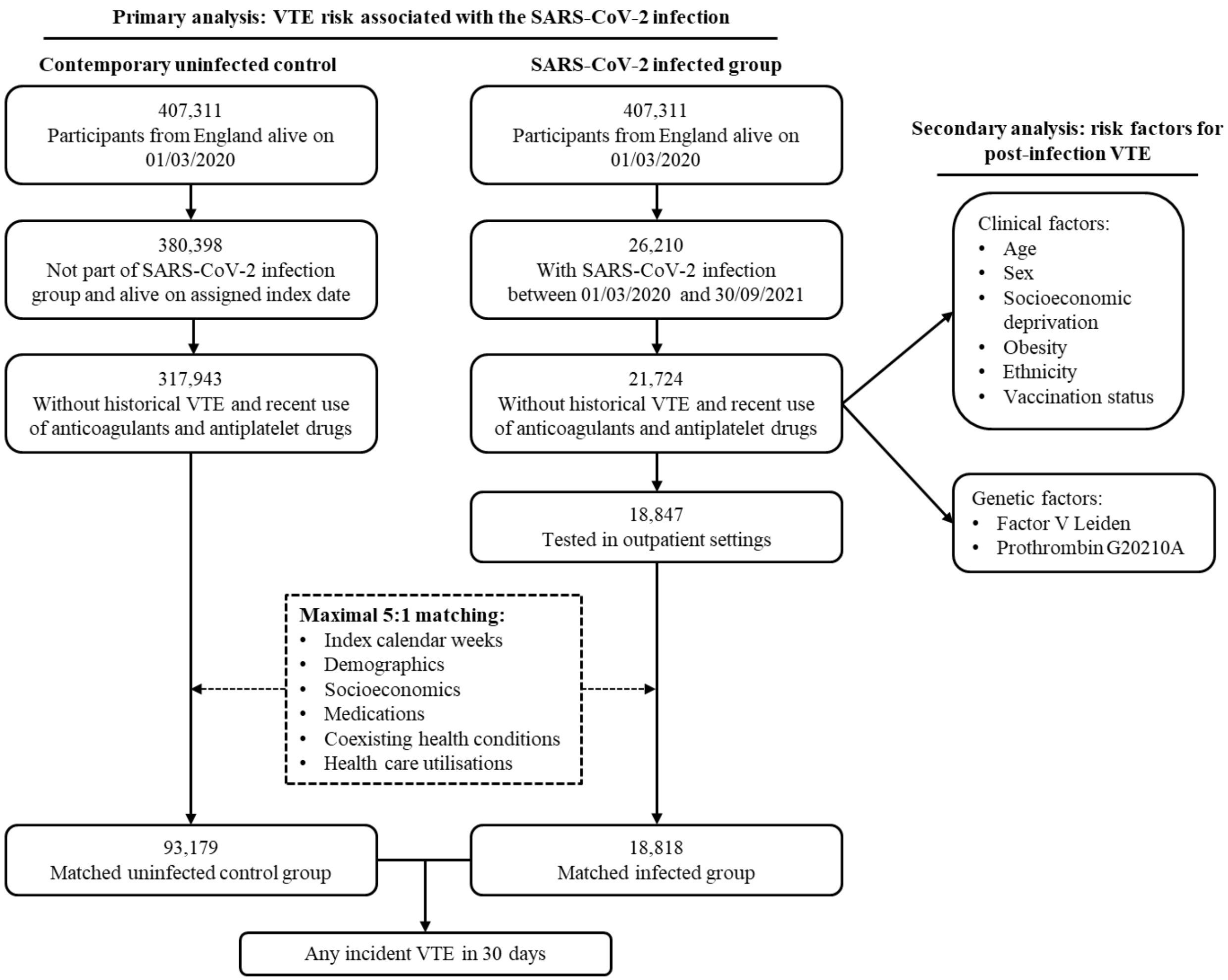

© 2022 Xie J et al. *JAMA Internal Medicine.*

eFigure 2: The distribution of infected and matched non-infected persons over the study period.

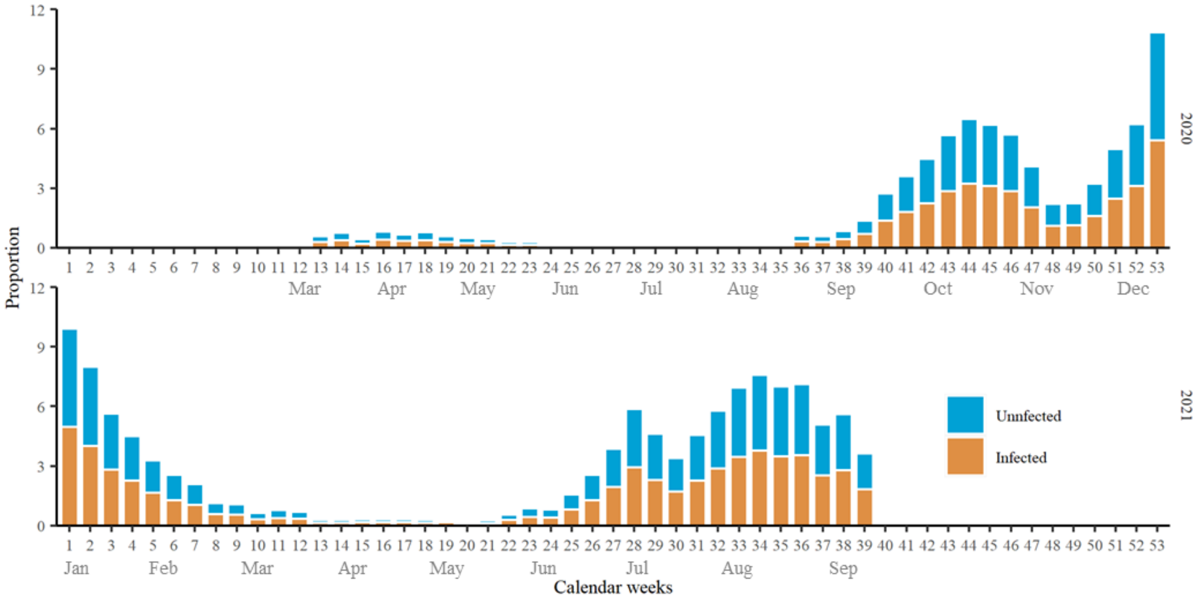

eTable 1: Baseline characteristics of participants stratified by the VTE outcome during the follow-up period.

	People without VTE outcome	People with VTE outcome	SMD
Number	336653	137	
Age, mean (sd)	67.75 (8.08)	69.61 (7.87)	0.233
Sex, male (%)	140218 (41.7)	75 (54.7)	0.264
Ethnicity, White (%)	314064 (93.3)	121 (88.3)	0.173
Index of multiple deprivation, mean (sd)	17.19 (13.59)	21.76 (15.70)	0.311
Body mass index, mean (sd)	27.08 (4.63)	29.19 (5.36)	0.423
**Vaccination status (%)**			
Not or partially vaccinated	206624 (61.4)	106 (77.4)	0.352
**Recent medications (%)**			
Lipid lowering drugs	91756 (27.3)	42 (30.7)	0.075
RAS inhibitors	61092 (18.1)	36 (26.3)	0.197
Other anti-hypertensives	27305 (8.1)	23 (16.8)	0.265
Proton pump inhibitors	83280 (24.7)	63 (46.0)	0.456
Diabetes medicines	17208 (5.1)	12 (8.8)	0.144
Antidepressants	46470 (13.8)	28 (20.4)	0.177
Systemic glucocorticoids	15184 (4.5)	25 (18.2)	0.443
Immunosuppressants	3739 (1.1)	5 (3.6)	0.167
Antineoplastic agents	192 (0.1)	0 (0.0)	0.034
**Recent orthopaedic surgery (%)**	6382 (1.9)	7 (5.1)	0.175
**Recent hospital admissions, mean (sd)**	0.29 (1.51)	1.56 (3.68)	0.452
**Coexisting conditions (%)**			
Fracture	60922 (18.1)	32 (23.4)	0.13
Fall	25425 (7.6)	19 (13.9)	0.205
Cancer	32719 (9.7)	28 (20.4)	0.303
Malignant cancer	1179 (0.4)	3 (2.2)	0.165
Diabetes (uncomplicated)	24731 (7.3)	15 (10.9)	0.125
Diabetes (end-organ damage)	7122 (2.1)	7 (5.1)	0.161
Congestive heart failure	1753 (0.5)	2 (1.5)	0.095
Myocardial infarction	691 (0.2)	1 (0.7)	0.077
Cerebrovascular disease	2637 (0.8)	1 (0.7)	0.006
Peripheral vascular disease	1426 (0.4)	2 (1.5)	0.107
Liver disease (mild)	1586 (0.5)	1 (0.7)	0.034
Liver disease (moderate to severe)	653 (0.2)	0 (0.0)	0.062
COPD	53121 (15.8)	22 (16.1)	0.008
Chronic kidney disease	13529 (4.0)	9 (6.6)	0.114
Peptic ulcer	6667 (2.0)	8 (5.8)	0.2
Rheumatoid arthritis	8864 (2.6)	10 (7.3)	0.216
Dementia	2105 (0.6)	2 (1.5)	0.082
Hemiplegia	257 (0.1)	0 (0.0)	0.039
AIDS	326 (0.1)	0 (0.0)	0.044

SMD: Standardised mean difference, COPD: Chronic obstructive pulmonary disease, AIDS: Acquired immune deficiency syndrome

eTable 2: Hazard ratio of clinical risk factors for venous thromboembolism amongst the infected and uninfected groups.

	Crude hazard ratio (95% CI)		Adjusted hazard ratio (95% CI)	
	**Among infected cohort**	**Among uninfected cohort**	**Among infected cohort**	**Among uninfected cohort**
Age (per 10-year increase)	1.93 (1.56 to 2.39)		1.87 (1.50 to 2.33)	
Sex (Male vs Female)	1.69 (1.31 to 2.19)		1.69 (1.30 to 2.19)	
Obesity (BMI >=30 vs <30)	2.08 (1.45 to 2.97)		1.83 (1.28 to 2.61)	
Socioeconomic status (Higher 50% IMD vs lower 50%)	1.41 (0.99 to 2.02)		1.21 (0.83 to 1.78)	
Ethnicity (Other ethnic vs White)	1.41 (0.91 to 2.17)		1.18 (0.74 to 1.88)	
Vaccination (Not or partial vs Full)	5.19 (2.86 to 9.42)	0.88 (0.66 to 1.17)	5.50 (3.00 to 10.08)	1.07 (0.80 to 1.42)

eTable 3: The frequency of risk variants for inherited thrombophilia.

	Inherited thrombophilia (any of the two SNPs)	Factor V Leiden	Prothrombin G20210A
All UKBB participants (407,311)	6.26%	4.43%	2.03%
Infected participants (21,724)	6.11%	4.32%	1.86%

eTable 4: Baseline characteristics amongst SARS-CoV-2 infected participants stratified by the inherited thrombophilia.

	**People without inherited thrombophilia**	**People with inherited thrombophilia**	**SMD**
Number	19768	1287	
Age, mean (sd)	64.82 (8.25)	64.82 (8.32)	0.001
Sex, male (%)	8804 (44.5)	555 (43.1)	0.028
Ethnicity, White (%)	16560 (83.8)	1131 (87.9)	0.118
Index of multiple deprivation, mean (sd)	19.97 (14.49)	19.48 (14.71)	0.034
Body mass index, mean (sd)	27.78 (4.88)	27.91 (5.03)	0.026
**Vaccination status (%)**			
Not or partially vaccinated	12442 (62.9)	802 (62.3)	0.013
**Recent medications (%)**			
Lipid lowering drugs	4744 (24.0)	316 (24.6)	0.013
RAS inhibitors	3383 (17.1)	232 (18.0)	0.024
Other anti-hypertensives	1512 (7.6)	92 (7.1)	0.019
Proton pump inhibitors	5370 (27.2)	341 (26.5)	0.015
Diabetes medicines	1210 (6.1)	70 (5.4)	0.029
Antidepressants	3277 (16.6)	228 (17.7)	0.03
Systemic glucocorticoids	1082 (5.5)	68 (5.3)	0.008
Immunosuppressants	220 (1.1)	16 (1.2)	0.012
Antineoplastic agents	19 (0.1)	0 (0.0)	0.044
**Recent orthopaedic surgery (%)**	451 (2.3)	27 (2.1)	0.013
**Recent hospital admissions, mean (sd)**	0.41 (2.30)	0.36 (1.33)	0.028
**Coexisting conditions (%)**			
Fracture	3481 (17.6)	215 (16.7)	0.024
Fall	1542 (7.8)	80 (6.2)	0.062
Cancer	1576 (8.0)	105 (8.2)	0.007
Malignant cancer	91 (0.5)	3 (0.2)	0.039
Diabetes (uncomplicated)	1614 (8.2)	97 (7.5)	0.023
Diabetes (end-organ damage)	455 (2.3)	29 (2.3)	0.003
Congestive heart failure	113 (0.6)	8 (0.6)	0.006
Myocardial infarction	51 (0.3)	2 (0.2)	0.023
Cerebrovascular disease	164 (0.8)	8 (0.6)	0.025
Peripheral vascular disease	65 (0.3)	2 (0.2)	0.035
Liver disease (mild)	93 (0.5)	7 (0.5)	0.010
Liver disease (moderate to severe)	54 (0.3)	3 (0.2)	0.008
COPD	3358 (17.0)	206 (16.0)	0.026
Chronic kidney disease	737 (3.7)	58 (4.5)	0.039
Peptic ulcer	431 (2.2)	25 (1.9)	0.017
Rheumatoid arthritis	541 (2.7)	32 (2.5)	0.016
Dementia	253 (1.3)	20 (1.6)	0.023
Hemiplegia	15 (0.1)	0 (0.0)	0.039
AIDS	19 (0.1)	0 (0.0)	0.044

SMD: Standardised mean difference, COPD: Chronic obstructive pulmonary disease, AIDS: Acquired immune deficiency syndrome

eMethods:

ICD-10 codes for the identification of venous thromboembolism.

"I26"	Pulmonary embolism
"I260"	Pulmonary embolism with mention of acute cor pulmonale
"I269"	Pulmonary embolism without mention of acute cor pulmonale
"I801"	Phlebitis and thrombophlebitis of femoral vein
"I802"	Phlebitis and thrombophlebitis of other deep vessels of lower extremities
"I803"	Phlebitis and thrombophlebitis of lower extremities, unspecified
"I81"	Portal vein thrombosis
"I82"	Other venous embolism and thrombosis
"I820"	Budd-Chiari syndrome
"I822"	Embolism and thrombosis of vena cava
"I823"	Embolism and thrombosis of renal vein
"I828"	Embolism and thrombosis of other specified veins
"I829"	Embolism and thrombosis of unspecified vein

Summary statistics for 297 candidate SNPs used for constructing the polygenic risk score for VTE.

**Chr:Pos**	rsid	**Effect Allele**	**Beta**
1:11910677	rs632793	G	0.0466
1:150388318	rs698915	A	0.0561
1:166533517	rs764024	T	0.0502
1:168406710	rs111414961	A	0.1183
1:168500034	rs72703796	G	0.2014
1:168572720	rs72705895	T	0.1313
1:168577239	rs12567872	T	0.0893
1:168619548	rs1933116	T	0.1724
1:168673803	rs611769	C	0.0562
1:168714137	rs10918970	T	0.0572
1:168729611	rs12117978	A	0.2166
1:168812398	rs34258243	G	0.1015
1:168889373	rs78516619	G	0.6841
1:168909952	rs7526462	T	0.0714
1:168937072	rs113123846	G	0.4417
1:168960286	rs1322487	A	0.046
1:168969254	rs35990973	A	0.1057
1:169012124	rs1320969	G	0.0478
1:169014610	rs116140155	A	0.2498
1:169031755	rs113079063	T	0.2356
1:169038856	rs12747018	T	0.0717
1:169064630	rs1200118	G	0.1111
1:169070213	rs1892091	C	0.0526
1:169079419	rs1358714	A	0.0444
1:169086013	rs2143289	T	0.2335
1:169107377	rs7520186	T	0.0873
1:169276816	rs10732287	T	0.0916
1:169316610	rs10158131	G	0.0673
1:169318242	rs72702145	A	0.1342
1:169463296	rs147474835	G	0.12
1:169463519	rs4656683	T	0.1781

**Chr:Pos**	rsid	**Effect Allele**	**Beta**
1:169486141	rs966751	G	0.1142
1:169511555	rs6032	T	0.1351
1:169593113	rs3917862	G	0.2198
1:169620673	rs12075684	G	0.0534
1:169659128	rs185120584	T	0.5696
1:170051841	rs115476742	C	0.0857
1:170080014	rs12128208	C	0.1373
1:170174298	rs12144655	A	0.0893
1:181031783	rs3936939	A	0.0458
1:201882277	rs2644120	C	0.058
1:207282149	rs2842700	A	0.1138
1:207285043	rs577695638	C	0.1708
1:230417394	rs3088075	T	0.0798
1:248028780	rs79322592	A	0.0517
1:248039451	rs3811444	C	0.0513
1:9341786	rs677665	T	0.0521
2:127934102	rs10198483	A	0.0559
2:127979335	rs111736896	A	0.0717
2:128133076	rs35293119	G	0.0442
2:128175875	rs1799809	G	0.072
2:128187428	rs67495946	C	0.0631
2:128398892	rs67076363	A	0.0733
2:161845453	rs75145714	C	0.1264
2:178238316	rs4893934	G	0.0449
2:198894550	rs535527575	G	0.1319
2:198946551	rs6434955	G	0.0587
2:68530180	rs13013670	T	0.0485
2:68619981	rs1867312	C	0.0585
3:126238371	rs11721316	A	0.061
3:126276710	rs12636448	C	0.0456
3:150872174	rs75347181	A	0.1323
3:150880908	rs3116549	C	0.0522
3:185234111	rs115110838	T	0.0649
3:194787481	rs116764841	T	0.0661
3:36140951	rs113114306	T	0.1047
3:39240559	rs7622284	T	0.0741
3:6084766	rs11712865	G	0.0799
3:77048575	rs6799348	G	0.1488
3:88354842	rs1841009	T	0.0567
3:89250573	rs7374904	A	0.0751
3:89679389	rs141912156	T	0.1656
3:90390344	rs9858006	A	0.0465
3:93518545	rs139156297	T	0.1588
3:93580976	rs9290378	T	0.0509
3:94209055	rs9290227	T	0.0849
4:155251071	rs116333064	A	0.1157
4:155424231	rs4323084	T	0.1012
4:155432944	rs4547780	G	0.064
4:155450158	rs13435192	T	0.1124
4:155474683	rs115999709	G	0.1456
4:155494926	rs79726896	G	0.1598

**Chr:Pos**	rsid	**Effect Allele**	**Beta**
4:155513276	rs2070008	T	0.0975
4:155515486	rs1984906	G	0.0671
4:155538470	rs13130318	G	0.1984
4:155542926	rs59234924	G	0.1077
4:155544958	rs72681247	T	0.2163
4:155553541	rs79201245	C	0.1462
4:187114479	rs10866290	C	0.0464
4:187130620	rs72646294	A	0.1947
4:187136519	rs113435394	C	0.2091
4:187161283	rs551251712	C	0.2385
4:187169469	rs2203111	G	0.0901
4:187178014	rs3775303	T	0.1486
4:187180115	rs4253333	G	0.0494
4:187190285	rs925452	A	0.2681
4:187196853	rs4253414	C	0.1544
4:187200550	rs56810541	T	0.1975
4:187205929	rs4253425	C	0.2399
4:187213360	rs72712610	A	0.1509
4:187219599	rs56379917	A	0.1638
4:187220894	rs13126546	T	0.1142
4:187234735	rs72712626	G	0.1365
4:187240280	rs13137269	T	0.0617
4:187267791	rs75440104	C	0.1177
4:187347217	rs7685922	C	0.054
4:79917638	rs140303646	T	0.218
5:143119511	rs325247	C	0.0439
5:172785895	rs12234072	G	0.0661
5:32831939	rs12656497	T	0.0438
5:38708554	rs16867574	C	0.059
5:63916834	rs4700642	A	0.0491
5:75992254	rs56347914	C	0.0843
6:147701133	rs9373523	G	0.06
6:169633335	rs11759438	C	0.0436
6:25531133	rs214057	C	0.0446
6:28436060	rs2531815	T	0.047
6:29894392	rs1627764	G	0.0525
6:30639412	rs3094094	A	0.0793
6:31092767	rs3095304	T	0.0634
6:31239869	rs2074492	T	0.051
7:151028181	rs11981586	C	0.0657
7:157763424	rs1347390	G	0.0515
8:102881195	rs118105926	A	0.1379
8:106573309	rs7341574	T	0.0452
8:106590705	rs4541868	C	0.0876
8:108291878	rs4236786	C	0.0512
8:108340982	rs7004172	G	0.051
8:108347806	rs6991048	T	0.0904
8:27810577	rs2685413	G	0.0608
8:27820792	rs10087301	A	0.0632
8:30265541	rs117564659	G	0.1314
8:53204323	rs138757339	C	0.2102

**Chr:Pos**	rsid	**Effect Allele**	**Beta**
8:78572803	rs17383689	G	0.0833
8:87143573	rs7812868	C	0.0621
9:116253293	rs189064188	T	0.0804
9:124362398	rs146383320	T	0.1278
9:135985796	rs3761824	C	0.0538
9:136025460	rs3888561	C	0.0559
9:136031918	rs7027827	A	0.0729
9:136056956	rs10793953	G	0.0496
9:136062437	rs10441806	C	0.105
9:136069931	rs7039497	G	0.1081
9:136077004	rs11244032	C	0.0837
9:136080512	rs149189328	C	0.2669
9:136081319	rs11244035	T	0.2223
9:136098498	rs28470788	T	0.0723
9:136121303	rs7855466	T	0.1054
9:136124590	rs78755596	A	0.2855
9:136128731	rs11244051	A	0.384
9:136144593	rs66697526	G	0.1399
9:136145404	rs9411377	A	0.308
9:136152070	rs8176634	G	0.1117
9:136152722	rs8176630	C	0.1382
9:136156064	rs55988407	G	0.1664
9:136156230	rs78590974	T	0.2221
9:136157037	rs557317	A	0.1499
9:136177993	rs4962043	G	0.0607
9:136184782	rs9411395	G	0.1408
9:136184985	rs11791119	T	0.1052
9:136185324	rs11789139	G	0.1669
9:136193356	rs76771223	G	0.1431
9:136212168	rs117119759	A	0.2099
9:136226421	rs141397052	G	0.2259
9:136240304	rs3124755	C	0.1278
9:136255149	rs62575992	C	0.0967
9:136268084	rs3124747	A	0.0757
9:136270538	rs41302673	G	0.242
9:136277854	rs2285488	G	0.1007
9:136296530	rs149181677	T	0.2461
9:136311017	rs652600	A	0.0992
9:136323826	rs3094373	A	0.2432
9:136343647	rs3094326	G	0.1027
9:136359182	rs736418	G	0.0556
9:136365146	rs28615587	T	0.078
9:136382716	rs9802874	A	0.0636
9:136390015	rs13300181	A	0.0616
9:136397195	rs11507716	T	0.0802
9:136509514	rs1611128	G	0.0495
10:121010256	rs10886430	G	0.1289
10:32397591	rs211416	T	0.0684
10:45632668	rs2211163	A	0.0724
10:71144324	rs2305196	A	0.0555
10:71148728	rs12416320	G	0.1553

**Chr:Pos**	rsid	**Effect Allele**	**Beta**
10:71153882	rs3793846	T	0.0572
10:71181371	rs36054387	C	0.0729
10:71215107	rs137936874	G	0.2231
10:71218059	rs10998791	A	0.0677
10:71245276	rs78707713	T	0.2414
10:71262048	rs1665581	G	0.0771
10:71333897	rs12785008	C	0.0691
10:71346272	rs12783163	A	0.0596
10:76189250	rs140438685	A	0.2024
10:80898969	rs1769758	G	0.0436
10:96011865	rs1547643	G	0.0476
11:126300537	rs11600151	T	0.0793
11:32967270	rs563259534	T	0.1048
11:33247621	rs2061997	T	0.049
11:46559730	rs11038913	T	0.1126
11:46745003	rs5896	T	0.0668
11:46893108	rs2306029	T	0.0641
11:46896126	rs72897640	T	0.0672
11:47373425	rs2856656	C	0.3976
11:47794348	rs34953939	A	0.0931
11:48865680	rs369876615	C	0.1184
11:50242788	rs117653193	T	0.1049
11:51282525	rs11606922	C	0.1115
11:55436134	rs72910502	C	0.1161
11:56526894	rs543926510	C	0.1339
11:56735815	rs75348906	A	0.0695
11:56875074	rs141798115	T	0.2207
11:61489705	rs198428	A	0.0464
11:61571348	rs174548	C	0.0842
11:61621611	rs73487492	A	0.1034
11:73283937	rs12274057	T	0.0751
12:104147207	rs3751198	G	0.0491
12:111932800	rs7137828	C	0.0424
12:123861452	rs28413626	G	0.0739
12:32844798	rs61926202	A	0.2109
12:39156743	rs137870902	T	0.2329
12:54734289	rs11170877	A	0.0696
12:6071943	rs139727584	C	0.1076
12:6150824	rs183356	A	0.1405
12:6160614	rs7135039	T	0.0781
12:6170645	rs78915411	G	0.0823
13:113787459	rs3211752	G	0.06
13:113808274	rs12858483	G	0.0588
14:100108918	rs12886724	G	0.0501
14:26690604	rs77398404	T	0.0794
14:66082793	rs2229678	C	0.1842
14:83281618	rs112089121	G	0.0752
14:92217670	rs61988257	A	0.0603
14:94838142	rs112635299	T	0.1463
15:43757184	rs190543502	T	0.1973
15:43911751	rs115384559	T	0.3853

**Chr:Pos**	rsid	**Effect Allele**	**Beta**
15:44880783	rs148770227	C	0.1837
15:65114833	rs35204896	G	0.0738
15:66430422	rs74245462	T	0.0503
15:96125226	rs17502085	A	0.0525
16:1405044	rs116468525	A	0.2059
16:81840709	rs34603417	A	0.0456
16:81844607	rs9937779	C	0.0568
16:81870969	rs12445050	T	0.1248
16:81874200	rs61374069	A	0.0524
16:81896523	rs55909816	C	0.0437
16:81902990	rs4889419	G	0.0575
16:81915832	rs11150422	G	0.053
16:81971403	rs1071644	C	0.0475
16:81976177	rs16956040	C	0.0613
16:89265466	rs12926888	A	0.0449
17:1966457	rs1048483	T	0.0616
17:2172753	rs216181	A	0.072
17:43758898	rs57222984	G	0.0522
17:67081278	rs77542162	G	0.1932
17:7785590	rs78209469	T	0.0867
17:8393900	rs2270744	C	0.0447
18:74465347	rs62112094	A	0.059
18:75283432	rs2032276	G	0.0435
18:8800723	rs631126	C	0.0579
19:10639312	rs8100818	T	0.0483
19:10659971	rs11668544	G	0.1314
19:10688153	rs187758170	A	0.1465
19:10734951	rs8110479	C	0.1131
19:10741622	rs12710257	G	0.1164
19:10898413	rs8107372	T	0.0515
19:17004049	rs1054533	T	0.0451
19:33896432	rs4805881	C	0.0544
19:3464793	rs142170418	C	0.1574
19:45426792	rs141622900	A	0.1007
19:46268902	rs2341097	T	0.0502
19:49241006	rs12981072	C	0.0469
19:55536595	rs1613662	A	0.0818
19:7832001	rs874492	A	0.0481
20:22672552	rs6137727	A	0.0534
20:22938940	rs62204096	A	0.1565
20:23000653	rs6076004	T	0.0472
20:23077117	rs149439892	A	0.1527
20:23170450	rs34397775	A	0.084
20:23182559	rs6083037	A	0.0798
20:32485961	rs6087538	C	0.0585
20:32523172	rs6059574	G	0.0479
20:33434252	rs56244533	A	0.0567
20:33435161	rs17092148	G	0.0612
20:33451060	rs75627267	T	0.1209
20:33587569	rs551986443	C	0.1488
20:33612647	rs564783003	C	0.1488

**Chr:Pos**	rsid	**Effect Allele**	**Beta**
20:33733180	rs2050652	G	0.0564
20:33762035	rs2069946	C	0.1255
20:33772243	rs6060288	A	0.1178
20:33858772	rs6058218	G	0.0552
20:33895947	rs6058227	T	0.0813
20:34025983	rs143383	G	0.0482
20:34562935	rs185663249	A	0.1487
20:34712310	rs6141600	C	0.0482
22:42461918	rs2854827	G	0.0589
22:43107837	rs9620086	G	0.0609
22:44324730	rs738408	C	0.0566
1:169519049	rs6025	T	0.4031
11:46761055	rs1799963	A	0.2741
